# Comparative Analysis of Polyphenol Content and Antioxidant Activity of Different Parts of Five Onion Cultivars Harvested in Korea

**DOI:** 10.3390/antiox13020197

**Published:** 2024-02-04

**Authors:** Yena Kim, Young-Jun Kim, Youngjae Shin

**Affiliations:** 1 Department of Food Engineering, Dankook University, Cheonan, Chungnam 31116, Republic of Korea; kimyn0930m@korea.kr; 2 Department of Food Science and Biotechnology, Seoul National University of Science and Technology, Seoul 01811, Republic of Korea

**Keywords:** onion, by-product, polyphenol, antioxidants, vitamin C

## Abstract

Onions are typically consumed as the bulb, but the peel and root are discarded as by-products during processing. This study investigated the potential functional use of these by-products by analyzing the polyphenols, antioxidant compounds, and antioxidant activity contained in onions. In this study, the bulb, peel, and root of five onion cultivars (‘Tank’, ‘Bomul’, ‘Gujji’ ‘Cobra’, and ‘Hongbanjang’) harvested in Korea were investigated. Caffeic acid and quercetin were most abundant in the peel, whereas methyl gallate was the predominant polyphenol in the bulb. Both DPPH and ABTS radical scavenging activity were higher in onion peel and root than in the bulb. These findings suggest that onion peel and roots, which are often discarded, have abundant antioxidant substances and excellent antioxidant activity. This study provides basic data for the future use of onion peel and roots as functional ingredients with high added value.

## 1. Introduction

*Allium cepa* L., commonly referred to as onions, are perennial plants of the lily family. Their distinct flavor and aroma have led to their predominant use as seasoning vegetables [1]. Phytochemicals, such as anthocyanins, flavonoids, polyphenols, and carotenoids, abundantly found in fruits and vegetables, are known to have beneficial effects in preventing oxidative stress-related aging, cardiovascular diseases, diabetes, and cancers [2,3]. Fruits and vegetables are high in water content and rich in dietary fiber, vitamins, and minerals, which can not only compensate for nutritional deficiencies in the main meal, but are also reported to reduce oxidative stress, improve blood lipid metabolism, lower blood pressure, increase insulin sensitivity, and help regulate body homeostasis [4]. According to onion color, white, yellow, and red onions are the most common types produced in many countries. Despite red onions being cultivated in comparatively smaller regions than yellow onions, their consumption is increasing among consumers who are aware of the health benefits of red onions [5]. Onions have been reported to have antioxidant effects on lipid peroxidation, which are mainly attributed to phenolic compounds, including flavonoids [6,7,8]. Onions contain quercetin, quercitrin, and rutin, and also the sulfur compounds allyl propyl disulfide and diallyl disulfide [9], which have antioxidant, anticancer, anti-diabetic, and anti-inflammatory effects. Quercetin, featuring 3′,4′ dihydroxy substitutions in the B ring and a conjugation linking the A and B rings, exhibits antioxidant capacities four times greater than Trolox, the vitamin E analogue. Onions are a good source of sugar, minerals, and vitamin C. Red onions contain anthocyanins, which are naturally occurring pigments that are widely distributed in red, purple, or blue-colored vegetables and are found not only in onions but also in red cabbage, lettuce, and eggplant [10]. Anthocyanins have been reported to possess bioactive effects such as antioxidative, anticancer, anti-inflammatory, inhibition of lipid peroxidation, and protection against DNA damage related to chronic diseases [6,11]. The outer layer of onions is typically discarded in industrial processing or home cooking, being deemed as environmental waste. Nevertheless, onion peel contains high levels of flavonoids, plant-derived dietary compounds known for their beneficial effects on human health, surpassing the concentrations found in the inner bulb of the onion that is commonly consumed [12]. Depending on countries, the by-products of onions, the peels and roots, may or may not be edible according to their respective food regulations. However, in South Korea, both the peel and roots are considered edible, and they are primarily utilized, often in the form of tea [13].

Studies comparing the antioxidant content and activity of onions’ bulbs and their by-product, the peel, have revealed that the total phenolic compound content was approximately 8.5 times higher in the peel than in the bulb, and the antioxidant activity was about 5 times greater in the peel [14]. Additionally, they reported that flavonol compounds, including quercetin, increase from the bulb to the peel or leaf portion, with the highest concentration found in the onion peel [1]. It has been shown that the by-products contain more bioactive compounds compared to the edible part. The bulb part of the onion is mainly consumed, and the amount of peel and root that is discarded continues to increase [15]. Therefore, there is a need to explore ways to utilize these by-products, but so far, research on onions has focused on the edible part of the onion, and research on the by-products is rare. In particular, there are no studies on the antioxidants and antioxidant activity of onion roots in the world. Therefore, this study aimed to analyze the antioxidant compounds and activity of five onions cultivated in Korea, divided into bulbs, peels, and roots. The goal was to obtain fundamental data to use the utilization of onion peels and roots as high value-added food ingredient containing abundant natural bioactive compounds.

## 2. Materials and Methods

### 2.1. Materials for Experiment

The yellow onions ‘Tank’, ‘Bomul’, and ‘Gujji’ used in this study were harvested from Muan Stevia Onion Farm, and the red onion ‘Cobra’ was harvested from Tongil Farm in Muan, Jeollanam-do, respectively. The other red onion ‘Hongbanjang’ was harvested from Seonghone Farm in Changnyeong, Gyeongsangnam-do. The onion samples were sorted into uniform sizes, divided into bulb, peel, and root, and washed under running water. After draining, they were frozen in liquid nitrogen at −196 °C and kept in a freezer at −20 °C for extraction of antioxidant compounds.

### 2.2. Sample Extraction

The frozen sample, 25 g in weight, was homogenized with 250 mL of 80% methanol using a blender (JB 3060, Braun Co., Kronberg, Germany) 3 times for 3 min each time. The homogenized solution was then filtered through a Whatman #2 filter paper (Whatman International Ltd., Kent, UK) using a vacuum filtration apparatus. The filtered solution was concentrated under vacuum using a rotary evaporator (N-1000, Eyela, Japan) at 45 °C and stored at −20 °C for subsequent analysis of antioxidant compounds and antioxidant activity.

### 2.3. Total Flavonoid Content

The total flavonoid content of the samples was measured using the colorimetric assay method [16]. A mixture of deionized water (4 mL) and sample extract (1 mL) was prepared, followed by the addition of 5% NaNO_2_ solution (0.3 mL). The mixture was vortexed and allowed to stand at room temperature for 5 min. Then, 0.3 mL of 10% AlCl_3_ was added, and the mixture was vortexed and allowed to react at room temperature for 6 min. Afterward, 1 M NaOH solution (2 mL) and distilled water (2.4 mL) were added to bring the total volume 10 mL, and the mixture was vortexed. The absorbance was measured at 415 nm using a spectrophotometer (Optizen POP, Mecasys, Daejeon, Republic of Korea). The standard curve was measured using quercetin, and the total flavonoid content was expressed as mg quercetin equivalents (QE)/100 g fresh weigh (FW).

### 2.4. Total Phenolic Content

The total phenolic content of the samples was measured using the Folin–Ciocalteu colorimetric assay method [16]. A mixture of deionized water (2.6 mL) and sample extract (0.2 mL) was prepared, followed by the addition of Folin–Ciocalteu’s phenol reagent (0.2 mL). The mixture was vortexed and allowed to stand at room temperature for 6 min. Then, 7% Na_2_CO_3_ solution (2 mL) was added, vortexed again, and the mixture was allowed to stand at room temperature in the dark for 90 min. The absorbance was then measured at 750 nm using a spectrophotometer. A standard calibration curve was constructed using gallic acid as a standard substance. The total phenolics content was expressed as mg gallic acid equivalents (GAE)/100 g FW.

### 2.5. Total Anthocyanin Content

The total anthocyanin content of the samples was determined using the pH differential method [16]. The extract was mixed with 0.025 M potassium chloride buffer (pH 1.0) and 0.4 M sodium acetate buffer (pH 4.5), and the absorbance was measured at 510 nm and 700 nm using a spectrophotometer. The total anthocyanin content was calculated using the following equation and expressed in units of mg cyanidin 3-O-glucoside equivalents (CGE)/100 g FW.

$$\mathrm{Total}\;\mathrm{anthocyanin}\;\mathrm{content}\;(\mathrm{mg}\;\mathrm{CGE}/100\;g\;\mathrm{FW})=\;\frac{\left(A\times \mathrm{MW}\times D\times 1000\right)}{\varepsilon \;}$$

A (absorbance value) = [(A_510 nm_-A_700 nm_)_pH 1.0_ − (A_510 nm_-A_700 nm_)_pH 4.5_]

MW (cyanidin 3-O-glucoside molecular weight) = 449.2

D = Dilution factor

*ε* (molar extinction coefficient of cyanidin 3-O-glucoside) = 26,000

### 2.6. Total Ascorbic Acid Content

The total ascorbic acid content of the samples was analyzed using the dinitrophenylhydrazine (DNPH) method of Terada et al. [17]. Five grams of homogenized samples were mixed with 100 mL of 6% metaphosphoric acid (buffer) and mixed well. The mixture was then centrifuged for 20 min at 15,000 rpm and filtered using a Whatman #1 filter paper. Then, 1 mL of the supernatant was mixed with 0.05 mL of 2% DCIP (2,6-Dichlorophenolindophenol) and allowed to react at room temperature for 1 h. Afterward, 1 mL of 2% thiourea was added, followed by the addition of 0.5 mL of 2% DNPH. The mixture was vortexed and allowed to react for 3 h at 60 °C. After cooling, 2.5 mL of 90% H_2_SO_4_ was slowly added to remove osazone. The absorbance was then measured at 540 nm using a spectrophotometer. A standard calibration curve was constructed using ascorbic acid as a standard substance. The total ascorbic acid content was expressed as mg/100 g FW.

### 2.7. Analysis of Phenolic Compounds by HPLC

For the polyphenol (protocatechuic acid, methyl gallate, caffeic acid, epicatechin, syringic acid, 4-methylcatechol, (-)-epicatechin gallate, ferulic acid, and rutin hydrate) content, the samples were analyzed using the method of Lee and Shin [18]. The sample extract was diluted using a dilution solution (KH_2_PO_4_:MeOH:D.W = 2:3:15). The diluted solution was filtered through a 0.45 μm syringe filter. The filtered samples were analyzed using HPLC (Agilent 1100 series, Agilent Technol., Wilmington, DE USA). The Eclipse XDB C-18 column (150 × 4.6 mm, 5 µm, Agilent Technol., Wilmington, DE USA) was operated at 40 °C. The HPLC mobile phase was 3% acetic acid (A) and methanol (B). The gradient conditions were 0–4 min, 100–90% A; 4–15 min, 90–45% A; 15–18 min, 45–100% A; and 18–23 min, 100% A. The detector was set to a wavelength of 280 nm at a flow rate of 0.9 mL/min. The 10 μL analysis sample was injected. The standard calibration curve was prepared by using the following standard substances: protocatechuic acid, methyl gallate, caffeic acid, epicatechin, syringic acid, 4-methylcatechol, (-)-epicatechin gallate, ferulic acid, and rutin hydrate with a retention time (RT) of 3.24, 8.94, 10.35, 10.86, 11,42, 11.68, 11.96, 13.67 and 14.97, respectively.

For quercetin-4′-O-glucoside and quercetin determination, the HPLC analytical conditions were adopted by Seo et al. [19]. Samples were analyzed using an UltiMate 3000 HPLC system (Thermo Fisher Scientific, Waltham, MA, USA) with a quaternary pump, autosampler, column compartment, and UV detector. Quercetin and quercetin-4′-O-glucoside were separated by applying a Capcell Pak C18 column (Osaka Soda, Osaka, Japan, 250 × 4.6 mm, 5 μm) at 40 °C. The mobile phase consisted of 0.1% formic acid in water (A) and methanol (B). The gradient conditions were 0–25 min, 80–40% A; 25–26 min, 40–0% A; 26–30 min, 0–40% A; 30–31 min, 40–80% A, equilibrated with 80% A for 7 min. The detector was monitored at 360 nm with a flow rate of 0.8 mL/min. The injection volume was 10 μL. Quercetin-4′-O-glucoside and quercetin were identified based on retention time of 17.49 min and 22.01 min, respectively. The analysis results were expressed as mg/100 g FW.

### 2.8. DPPH Radical Scavenging Activity

The antioxidant activity of the onion samples was measured using a modified DPPH assay method based on the methods described by Yang et al. [16]. A 100 μM DPPH solution was prepared using 2,2-diphenyl-1-picrylhydrazyl and 80% methanol, and diluted to achieve an absorbance of 0.63–0.67 at 517 nm wavelength. 50 μL of the sample extract was mixed with 2.95 mL of DPPH solution, and the mixture was incubated at room temperature in the dark for 30 min. The absorbance of the mixture was then measured at 517 nm using a spectrophotometer. A standard calibration curve was constructed using ascorbic acid as a standard. The DPPH radical scavenging activity was expressed as mg vitamin C equivalents (VCE)/100 g fresh weight (FW).

### 2.9. ABTS Radical Scavenging Activity

The antioxidant activity of the onion samples was measured using a modified ABTS assay method based on the method described by Yang et al. [16]. A 1 mM 2,2′-Azobis(2-amidinopropane) dihydrochloride (AAPH) and 2.5 mM ABTS were mixed with PBS buffer and allowed it to react at 70 °C for 40 min in a water bath. The solution was then diluted with PBS to achieve an absorbance of 0.63–0.67 at 734 nm wavelength. Then, 20 μL of the sample extract was mixed with 980 μL of ABTS solution, and the mixture was incubated at 37 °C for 10 min. The absorbance of the mixture was then measured at 734 nm using a spectrophotometer. A standard calibration curve was constructed using ascorbic acid as a standard. The ABTS radical scavenging activity was expressed as mg VCE/100 g FW.

### 2.10. Statistical Analysis

For the statistical analysis of each experimental result, the SPSS 20 program (SPSS Inc. Chicago, IL, USA) was used to perform the analysis of variance (ANOVA). Duncan’s multiple range test was used to test the significance of differences among the samples (*p* < 0.05). The data were expressed as the mean ± standard deviation from triplicate determination. The correlation between the mean values of each factor was expressed using Pearson’s correlation coefficient.

## 3. Results and Discussion

### 3.1. Total Flavonoid Content

The total flavonoid contents of onions are shown in Table 1. There was no significant difference in the total flavonoid content of the bulbs between the cultivars. The total flavonoid content of the peel was significantly higher in the ‘Bomul’ cultivar, at 2366.39 ± 67.37 mg QE/100 g. The total flavonoid content of the roots was measured from 69.73 ± 11.92 mg to 114.45 ± 18.00 mg QE/100 g FW. The total flavonoid content of the roots was significantly higher in the ‘Gujji’ cultivar. The total flavonoid content per part was higher in the peel and roots, which are by-products, than in the edible part, the bulb, with the peel having the highest content in all cultivars. In contrast to the edible onion bulb, onion peel exhibited the highest levels of phenolics and flavonoids [20,21]. Furthermore, the onion peels were noted for their significant flavonoid content, measuring 55.27 mg QE/g dw [22]. These differences are believed to be due to factors such as onion variety, growing region, and environmental conditions. A study by Jang and Lim [23] found that the total flavonoid content in onion bulbs was 19.67 µg/mL DW, while in the peel, it was 951.1 µg/mL DW. Onion peel exhibited approximately 48 times higher flavonoid content compared to the bulbs. Our results are consistent with these findings, as the total flavonoid content of peels was 50 to 150 times higher than that of flesh.

### 3.2. Total Phenolic Content

The total phenolic content of the bulbs was not significantly different between cultivars, as was the total flavonoid content (Table 1). The total phenolic content of the peels was significantly higher in the ‘Bomul’ cultivar, at 2487.56 ± 47.83 mg GAE/100 g FW, followed by the ‘Hongbanjang’ cultivar at 2035.56 ± 16.67 mg GAE/100 g FW. The total phenolic content of the roots was measured to be between 77.35 ± 2.39 mg and 103.28 ± 5.23 mg GAE/100 g FW, and was significantly higher in the ‘Gujji’ cultivar, as was the total flavonoid content. The total phenolic content in the inedible parts, peel, and roots, was significantly higher compared to the edible part, the bulb. According to a study by Lee et al. [24], the total phenolic content of onion bulbs was measured to be between 4.02 mg and 23.12 mg GAE/g DW, which is similar to the results of our study. A study by Lisanti et al. [25] also found that the total phenolic content of onion bulbs was between 4.71 ± 1.05 mg and 6.61 ± 0.87 mg GAE/g DW, which is also similar to our finding. The peel and skin of whole yellow onions grown in southern Sweden farms exhibited total phenol (mg GAE/g) content ranging from 27.8 to 51.1 and 54.7–68.2, respectively, on a dry weight basis [26]. Nevertheless, yellow peels from onions in the Galați local market, Romania, reported slightly higher total polyphenolic compounds at 97.28 mg GAE/g DW. These onion peels were also noted for their substantial flavonoid content, measuring 55.27 mg QE/g DW [22]. Extracts from onion peels in the Nigerian local market demonstrated 98.52 μg QE/mL total flavonoids and 664.30 μg/mL GAE total phenols [27].

### 3.3. Total Anthocyanin Content

The total anthocyanin content of the onions is shown in Table 1. Anthocyanins were only detected in red onions and not in yellow onions. The anthocyanin content of the bulbs of red onions was measured to be 12.50 ± 4.56 mg CGE/100 g FW for the ‘Cobra’ cultivar and 3.45 ± 0.32 mg CGE/100 g FW for the ‘Hongbanjang’ cultivar. The anthocyanin content of the ‘Cobra’ cultivar was significantly higher than that of the ‘Hongbanjang’ cultivar. The anthocyanin content of the peels of red onions was measured to be 254.82 ± 22.72 mg CGE/100 g FW for the ‘Cobra’ cultivar and 157.16 ± 21.22 mg CGE/100 g FW for the ‘Hongbanjang’ cultivar. Similar to the bulb, the anthocyanin content in the peel was significantly higher in the ‘Cobra’ cultivar compared to the ‘Hongbanjang’ cultivar. The total anthocyanin content was higher in the peels, which are by-products, than in the edible part, the bulbs, and was not detected in the roots. Albishi et al. [20] verified that red onion skin exhibited the highest anthocyanin content (10.04 mg anthocyanidin-3-glucoside/100 g), followed by pearl and yellow varieties. In contrast, the white varieties (0.06 mg anthocyanidin-3-glucoside/100 g) showed lower anthocyanin levels, potentially attributed to pigments present in colored onions contributing to higher anthocyanin content compared to white onion skin. According to a study by Slimestad et al. [28], the anthocyanin content of red onion bulbs accounts for 10% of the total flavonoid content, ranging from 39 to 240 mg/kg FW. This is similar to the results of our study.

### 3.4. Total Ascorbic Acid Content

The total ascorbic acid content of onions is shown in Table 2. The content in the bulbs was highest in the ‘Tank’ cultivar, measuring 21.51 ± 1.12 mg/100 g FW, which was approximately three times higher than the lowest content observed in the ‘Cobra’ cultivar. On the other hand, the ‘Cobra’ cultivar exhibited the highest content at 196.57 ± 13.54 mg/100 g FW in the peel, followed by the ‘Hongbanjang’ and ‘Tank’ cultivars. The total ascorbic acid content of the roots was highest in the ‘Gujji’ cultivar, at 4.16 ± 1.79 mg/100 g FW. The total ascorbic acid content was highest in the peel in all cultivars, followed by the bulb and the root. Jang et al. [29] reported that the total ascorbic acid content of onion bulbs was 6.27 mg/100 g FW, which is similar to our results for the ‘Bomul’ and ‘Cobra’ cultivars, but higher in other cultivars. This difference is attributed to variations in cultivars, growing environments, and extraction methods. The results of a study by Jang et al. [29] showed that the total ascorbic acid content of fresh red onions was 15 mg/100 g FW, which is similar to the results of ‘Hongbanjang’ cultivar. The total ascorbic acid content of dried red onion powder was 96 mg/100 g DW, which is also similar to the results of this study for the ‘Bomul’ and ‘Cobra’ cultivars. Geetha et al. [30] found that the total ascorbic acid content of onion peels was 0.49 mg/g FW and 0.75 mg/g FW, which is similar to the results of this study for the ‘Gujji’ and ‘Tank’ cultivars.

### 3.5. Phenolic Profile of the Samples

The polyphenol content of onions is provided in Table 3. The primary polyphenols identified in the bulb were methyl gallate, rutin hydrate, and quercetin-4′-O-glucoside. In the peel, the major polyphenols detected were caffeic acid, protocatechuic acid, and quercetin. For the roots, the primary polyphenols were caffeic acid and rutin hydrate. Quercetin and its glycosides, which are known to be the main polyphenols in onions, were detected in significant amounts in the peel, but only in trace amounts in the bulbs and roots. The onion’s skin, rich in quercetin, contributes to over 80% of the vegetable’s total flavonoid content. Furthermore, in comparison to the inner fleshy scales where 53% of total quercetin exists in its free form, the dry onion skins exhibit a more varied quercetin composition. Industrial processing of various onion parts preserves dietary fiber, fructans, and flavonoids, all of which offer health benefits. Simultaneously, these fragments from onion by-products can be utilized as an inexpensive, natural, and readily available source of beneficial components, particularly antioxidants [31].

In addition, methyl gallate was the most abundant polyphenol in the bulb, while caffeic acid was the most abundant in the peel. Methyl gallate, a major polyphenol in onion bulbs, is a phenolic compound found in many plants and is a methyl ester of gallic acid. Methyl gallate has a wide range of biological effects, including antioxidant, anti-inflammatory, anti-cancer, antibacterial, antiviral, and protection against DNA damage caused by oxidative stress [32]. In a study by Farg et al. [33], the methyl gallate content of spirulina, which has high antioxidant capacity and is used as a functional food, was measured to be 0.96 µg/mg DW, which is similar to, or slightly higher than the methyl gallate content of onion bulbs in our experiment. The major polyphenol in onion peel, caffeic acid, belongs to the hydroxycinnamic acid group and is abundant in various plant-derived foods such as fruits, vegetables, coffee, and tea. The caffeic acid content of coffee is reported to be in the range of 1.00 mg/g to 1.51 mg/g [34], which confirms that onion peels in this study contain more caffeic acid.

In the outer peel of onions, approximately 6.5% of quercetin is reported to be present relative to the peel’s weight, whereas the bulb contains about 0.01% quercetin relative to its weight, indicating a higher quercetin content in the outer peel [35]. Our results also confirmed that the peel has a higher quercetin content than the bulb. The main polyphenol in onion roots was also found to be caffeic acid, as in the peel while there have been numerous studies analyzing polyphenols in the edible part of onions, particularly the flesh, analyses of by-products like the peel have been limited. Notably, there is a lack of analysis for polyphenols in onion roots. Based on our study, caffeic acid was identified as the major polyphenol in both the peel and roots. Therefore, it is believed that the peel and roots of onions have high potential for use as functional ingredients.

### 3.6. Antioxidant Activity

Kumar et al. [36] state that onion waste, including skin and peel, are rich sources of total phenolic compounds and flavonoids, with quercetin and its derivatives being the main components contributing to their antioxidant activities. The total antioxidant activity results of onions are shown in Table 4. The DPPH radical scavenging activity of the peel was significantly higher in the ‘Hongbanjang’ and ‘Bomul’ cultivars, with values of 1259.41 ± 5.87 mg and 1176.34 ± 59.82 mg VCE/100 g FW, respectively. The DPPH radical scavenging activity of the roots was measured to be between 41.74 ± 3.71 mg VCE/100 g FW and 22.16 ± 1.12 mg VCE/100 g FW, depending on the cultivar. The DPPH radical scavenging activity of the roots was significantly highest in the ‘Gujji’ cultivar. The activity of the peel and roots was higher than that of the bulb, similar to the total flavonoids and total phenolics content results. The peel had significantly higher levels in all cultivars. The DPPH radical scavenging activity of the bulb showed significant differences between cultivars. The ‘Gujji’ cultivar exhibited over twice as high as the ‘Bomul’ cultivar.

The ABTS radical scavenging activity of the peel was also significantly higher in the ‘Bomul’ and ‘Hongbanjang’ cultivars, with values of 3007.07 ± 123.65 mg and 2988.44 ± 13.66 mg VCE/100 g FW, respectively. The ABTS radical scavenging activity of the roots was measured to be between 63.70 ± 2.26 mg VCE/100 g FW and 93.08 ± 3.83 mg VCE/100 g FW, depending on the cultivar. Similar to the DPPH radical scavenging activity, the ‘Gujji’ cultivar exhibited the highest ABTS radical scavenging activity, while the ‘Tank’ cultivar showed significantly lower activity. The ABTS radical scavenging activity of the bulb was lower than that of the roots in all cultivars. Many studies have reported that by-products like the peel exhibit higher antioxidant activity than the edible part [37], and in this study, the ABTS radical scavenging activity, similar to the results of total flavonoids and total phenolic contents, was higher in the peel and roots compared to the edible part, the bulb.

### 3.7. Correlation Analysis

**Figure 1:**
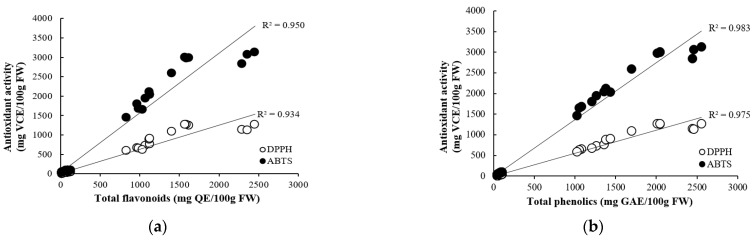
Correlation between antioxidant concentrations ((**a**) flavonoids and (**b**) phenolics) and activities (DPPH and ABTS radical scavenging activities).

The correlation between antioxidant compounds and antioxidant activity in onions is shown in Figure 1. The total flavonoids content in onions showed a strong positive correlation with DPPH radical scavenging activity (R^2^ = 0.934) and ABTS radical scavenging activity (R^2^ = 0.950). Total phenolics content also showed a strong positive correlation with DPPH radical scavenging activity (R^2^ = 0.975) and ABTS radical scavenging activity (R^2^ = 0.983). In a study by Zhang et al. [10], the correlation between total flavonoids and antioxidant capacity (DPPH, ABTS) in onion bulbs was very strong, with r = 0.926 and r = 0.920, respectively, and the correlation between total phenolics and antioxidant capacity (DPPH, ABTS) was also strong, with r = 0.764 and r = 0.787, respectively. Phenolic compounds were observed to be the major contributors to the antioxidant activity in fruits and vegetables. The increase in antioxidant activity in plant samples may be related to the release of more bound phenolic compounds from the samples [38,39].

## 4. Conclusions

The total flavonoids and total phenolics content of onion peel and roots were significantly higher than that of the bulb. Quercetin, which is known to be a major polyphenol in onions, was found in bulb and peel, but was detected in trace amounts in roots. The total anthocyanin content of red onion cultivars was significantly higher in ‘Cobra’ than in ‘Hongbanjang’ in both bulb and peel. The findings of this study suggest that onion peel and root have promising potential for application as functional materials, owing to their rich content of antioxidant compounds and superior antioxidant activity.

## Figures and Tables

**Table 1:** Total flavonoids, total phenolics, and total anthocyanin contents (mean ± SD in mg/100 g FW) of five onion (*Allium cepa* L.) cultivars and their parts (bulb, peel, and root).

		Tank	Bomul	Gujji	Cobra	Hongbanjang
Total flavonoids	Bulb	16.84 ± 3.10 ^a^	15.09 ± 0.74 ^a^	18.65 ± 3.66 ^a^	18.17 ± 1.49 ^a^	20.34 ± 1.89 ^a^
	Peel	1055.00 ± 66.25 ^cd^	2366.39 ± 67.37 ^a^	947.50 ± 83.59 ^d^	1215.28 ± 132.50 ^c^	1590.28 ± 17.80 ^b^
	Root	85.15 ± 4.93 ^b^	85.60 ± 5.65 ^b^	114.45 ± 18.00 ^a^	69.73 ± 11.92 ^b^	77.43 ± 13.09 ^b^
Total phenolics	Bulb	54.54 ± 3.41 ^a^	50.97 ± 0.16 ^a^	57.08 ± 4.74 ^a^	49.52 ± 1.17 ^a^	52.56 ± 3.70 ^a^
	Peel	1280.00 ± 61.01 ^d^	2487.56 ± 47.83 ^a^	1054.67 ± 23.95 ^e^	1506.22 ± 138.08 ^c^	2035.56 ± 16.67 ^b^
	Root	77.35 ± 2.39 ^b^	91.64 ± 4.94 ^ab^	103.28 ± 5.23 ^a^	80.27 ± 9.48 ^b^	86.90 ± 9.19 ^b^
Total anthocyanin	Bulb	ND	ND	ND	12.50 ± 4.56 ^a^	3.45 ± 0.32 ^b^
	Peel	ND	ND	ND	254.82 ± 22.72 ^a^	157.16 ± 21.22 ^b^
	Root	ND	ND	ND	ND	ND

Results are the mean values ± standard deviation from three measurements (n = 3); means in the same row with superscript with different letters (a, b, c, d, and e) are significantly different at *p* < 0.05. ND: Not detected.

**Table 2:** Total ascorbic acid contents (mean ± SD in mg/100 g FW) of five onion (*Allium cepa* L.) cultivars and their parts.

	Tank	Bomul	Gujji	Cobra	Hongbanjang
Bulb	21.51 ± 1.12 ^a^	9.27 ± 4.93 ^b^	12.24 ± 1.43 ^ab^	7.23 ± 3.86 ^b^	18.38 ± 8.41 ^ab^
Peel	76.11 ± 19.39 ^b^	21.39 ± 3.36 ^c^	42.22 ± 5.71 ^c^	196.57 ± 13.54 ^a^	95.37 ± 7.63 ^b^
Root	1.66 ± 0.28 ^b^	1.92 ± 0.24 ^b^	4.16 ± 1.79 ^a^	2.18 ± 0.94 ^ab^	2.41 ± 0.14 ^ab^

Results are the mean values ± standard deviation from three measurements (n = 3); means in the same row with superscript with different letters (a, b, and c) are significantly different at *p* < 0.05.

**Table 3:** Polyphenol contents (mean ± SD in mg/100 g FW) of five onion (*Allium cepa* L.) cultivars and their parts (bulb, peel, and root).

		Methyl Gallate	Rutin Hydrate	Quercetin-4′-O-Glucoside	Quercetin	Ferulic Acid	Protocatechuic Acid	Caffeic Acid	Epicatechin	Total Sum
Bulb	Tank	5.69 ± 0.05 ^a^	0.66 ± 0.03 ^b^	0.56 ± 0.01 ^c^	0.02 ± 0.00 ^c^	0.14 ± 0.00 ^b^	0.09 ± 0.00 ^a^	ND	ND	7.16
	Bomul	5.13 ± 0.02 ^b^	0.65 ± 0.01 ^b^	0.57 ± 0.01 ^c^	0.02 ± 0.00 ^e^	0.16 ± 0.00 ^a^	0.04 ± 0.00 ^c^	ND	ND	6.57
	Gujji	5.04 ± 0.09 ^b^	0.82 ± 0.02 ^a^	0.81 ± 0.00 ^a^	0.02 ± 0.00 ^d^	0.17 ± 0.01 ^a^	0.04 ± 0.00 ^bc^	ND	ND	6.90
	Cobra	3.54 ± 0.06 ^d^	0.76 ± 0.03 ^a^	0.76 ± 0.01 ^b^	0.02 ± 0.00 ^b^	0.08 ± 0.00 ^c^	0.04 ± 0.00 ^b^	ND	ND	5.20
	Hongbanjang	3.77 ± 0.04 ^c^	0.70 ± 0.03 ^b^	0.81 ± 0.00 ^a^	0.04 ± 0.00 ^a^	0.14 ± 0.00 ^b^	0.09 ± 0.00 ^a^	ND	ND	5.55
Peel	Tank	ND	ND	0.73 ± 0.00 ^c^	25.57 ± 0.15 ^c^	1.69 ± 0.06 ^a^	71.03 ± 2.93 ^c^	120.44 ± 7.05 ^b^	20.51 ± 0.59 ^c^	239.97
	Bomul	ND	ND	0.21 ± 0.01 ^e^	25.05 ± 0.50 ^c^	0.59 ± 0.02 ^e^	47.16 ± 0.08 ^e^	64.60 ± 0.30 ^c^	12.83 ± 0.36 ^e^	150.44
	Gujji	ND	ND	0.25 ± 0.00 ^d^	28.61 ± 0.36 ^b^	0.89 ± 0.02 ^c^	55.70 ± 0.28 ^d^	67.20 ± 0.30 ^c^	16.93 ± 0.14 ^d^	169.58
	Cobra	ND	ND	1.15 ± 0.01 ^b^	22.35 ± 0.24 ^d^	0.80 ± 0.01 ^d^	77.02 ± 0.23 ^b^	121.71 ± 1.14 ^b^	25.00 ± 0.55 ^b^	248.03
	Hongbanjang	ND	ND	1.89 ± 0.04 ^a^	39.48 ± 0.75 ^a^	1.09 ± 0.00 ^b^	98.58 ± 0.12 ^a^	214.30 ± 0.49 ^a^	35.11 ± 0.22 ^a^	390.45
Root	Tank	0.66 ± 0.01 ^b^	1.39 ± 0.05 ^d^	0.01 ± 0.00 ^d^	0.01 ± 0.00 ^d^	0.72 ± 0.03 ^d^	1.03 ± 0.04 ^e^	0.17 ± 0.01 ^e^	ND	3.99
	Bomul	1.21 ± 0.02 ^a^	3.67 ± 0.09 ^a^	0.01 ± 0.00 ^d^	0.01 ± 0.00 ^d^	1.67 ± 0.00 ^a^	1.97 ± 0.03 ^b^	0.50 ± 0.01 ^d^	ND	9.04
	Gujji	0.32 ± 0.00 ^d^	2.12 ± 0.07 ^c^	0.12 ± 0.00 ^a^	0.66 ± 0.00 ^a^	1.35 ± 0.03 ^c^	1.17 ± 0.05 ^d^	2.56 ± 0.11 ^c^	ND	8.30
	Cobra	0.27 ± 0.01 ^e^	0.32 ± 0.01 ^e^	0.04 ± 0.00 ^c^	0.14 ± 0.00 ^c^	1.32 ± 0.06 ^c^	1.43 ± 0.06 ^c^	4.49 ± 0.10 ^b^	ND	8.01
	Hongbanjang	0.54 ± 0.01 ^c^	2.63 ± 0.06 ^b^	0.08 ± 0.01 ^b^	0.16 ± 0.00 ^b^	1.50 ± 0.10 ^b^	2.48 ± 0.07 ^a^	4.66 ± 0.09 ^a^	ND	12.05

Results are the mean values ± standard deviation from three measurements (n = 3); means in the same column with superscript with different letters (a, b, c, d, and e) are significantly different at *p* < 0.05. ND: Not detected.

**Table 4:** Total antioxidant activities (mean ± SD in mg VCE/100 g FW) of five onion (Allium cepa L.) cultivars and their parts (bulb, peel, and root).

		Tank	Bomul	Gujji	Cobra	Hongbanjang
DPPH	Bulb	5.29 ± 1.35 ^ab^	3.75 ± 0.50 ^b^	8.71 ± 2.96 ^a^	7.54 ± 1.87 ^ab^	6.00 ± 1.58 ^ab^
	Peel	720.47 ± 37.24 ^c^	1176.34 ± 59.82 ^a^	620.78 ± 28.13 ^c^	959.31 ± 92.22 ^b^	1259.41 ± 5.87 ^a^
	Root	22.16 ± 1.12 ^c^	28.94 ± 2.17 ^b^	41.74 ± 3.71 ^a^	28.88 ± 3.54 ^b^	33.71 ± 3.16 ^b^
ABTS	Bulb	41.09 ± 4.76 ^a^	38.02 ± 0.27 ^a^	45.78 ± 6.93 ^a^	38.85 ± 3.89 ^a^	43.40 ± 3.91 ^a^
	Peel	1924.98 ± 100.42 ^c^	3007.07 ± 123.65 ^a^	1594.42 ± 96.18 ^d^	2242.43 ± 248.13 ^b^	2988.44 ± 13.66 ^a^
	Root	63.70 ± 2.26 ^c^	81.79 ± 5.46 ^ab^	93.08 ± 3.83 ^a^	78.39 ± 6.26 ^b^	84.85 ± 6.76 ^ab^

Results are the mean values ± standard deviation from three measurements (n = 3); means in the same row with superscript with different letters (a, b, c, and d) are significantly different at *p* < 0.05.

## Data Availability

Data is contained within the article.
